# RNA Activation—A Novel Approach to Therapeutically Upregulate Gene Transcription

**DOI:** 10.3390/molecules26216530

**Published:** 2021-10-28

**Authors:** Choon Ping Tan, Laura Sinigaglia, Valentí Gomez, Joanna Nicholls, Nagy A. Habib

**Affiliations:** 1 MiNA Therapeutics Ltd., Translation & Innovation Hub, 84 Wood Lane, London W12 0BZ, UK; tan@minatx.com (C.P.T.); laura@minatx.com (L.S.); valentin@minatx.com (V.G.); joanna@minatx.com (J.N.); 2 Department of Surgery and Cancer, Imperial College London, Hammersmith Hospital, London W12 0NN, UK

**Keywords:** small activating RNA, saRNA, oligonucleotide therapeutics, RNA activation, cancer treatment, clinical trial, CEBPA

## Abstract

RNA activation (RNAa) is a mechanism whereby RNA oligos complementary to genomic sequences around the promoter region of genes increase the transcription output of their target gene. Small activating RNA (saRNA) mediate RNAa through interaction with protein co-factors to facilitate RNA polymerase II activity and nucleosome remodeling. As saRNA are small, versatile and safe, they represent a new class of therapeutics that can rescue the downregulation of critical genes in disease settings. This review highlights our current understanding of saRNA biology and describes various examples of how saRNA are successfully used to treat various oncological, neurological and monogenic diseases. MTL-CEBPA, a first-in-class compound that reverses CEBPA downregulation in oncogenic processes using CEBPA-51 saRNA has entered clinical trial for the treatment of hepatocellular carcinoma (HCC). Preclinical models demonstrate that MTL-CEBPA reverses the immunosuppressive effects of myeloid cells and allows for the synergistic enhancement of other anticancer drugs. Encouraging results led to the initiation of a clinical trial combining MTL-CEBPA with a PD-1 inhibitor for treatment of solid tumors.

## 1. RNA Activation Is a Novel Class of Therapeutics

The hallmark of healthy cellular function is a well-regulated transcriptional program which expresses the correct dosages of genes at the right space and at the right time. Dysregulation (overexpression and/or down regulation) of gene expression results in a broad range of diseases which include, but is not limited to cancer, autoimmunity, neurological disorders, developmental syndromes, diabetes, cardiovascular diseases and obesity [1]. Years of clinical research on siRNA (small interfering RNA) and ASO (antisense oligonucleotide) has resulted in the approval of drugs that inhibit overexpression of undesirable genes, but there remained a need for treatments that can specifically turn genes back on after downregulation from specific diseases. The solution to this demand presented itself in the mid-2000s when two research groups independently showed that short RNA oligos designed to target the promoter region of several genes increased the transcription of their intended mRNA above basal levels [2,3]. This mechanism is now termed RNA activation (RNAa).

Distinct from other gene replenishment modalities such as plasmids, virus vectors or mRNA, RNAa upregulates target expression with molecules that are orders of magnitude smaller in size, which lowers the overall research and CMC (chemistry, manufacturing and control) costs. Furthermore, unlike the introduction of aberrant foreign genetic material, RNAa leverage on the reversible upregulation of endogenous genes to bring about a safe and well-tolerated change at the cellular level. As most genes use promoters for transcription regulation, RNAa represents a versatile digital platform whereby therapeutic RNA sequences could be directly extracted from the human genomic database. These attributes make RNAa a highly attractive therapeutic modality. To date, many research groups have reported the success of applying RNAa to their particular disease models and the first drug to utilize RNAa is currently in a clinical trial.

## 2. Molecular Mechanisms of RNAa

The phenomenon of RNAa was described as early as 1969 by Britten and Davidson [4]. More recent publications report how endogenously produced small RNAs can activate the transcription of genes involved in stem cell differentiation [5] and how microRNAs could enhance viral replication [6]. In 2006, Li et al. tested exogenously administered synthetic small RNAs against the promoter regions of human E-cadherin, p21WAF1/CIP1 (p21) and VEGF. They showed the specific upregulation of the target mRNAs in vitro and termed these oligos as small activating RNAs (saRNAs) [3]. Later, studies confirmed that RNAa is an evolutionarily conserved mechanism present in possibly all eukaryotic organisms, from *Caenorhabditis elegans* to mammals [7,8,9,10,11].

**Figure 1:**
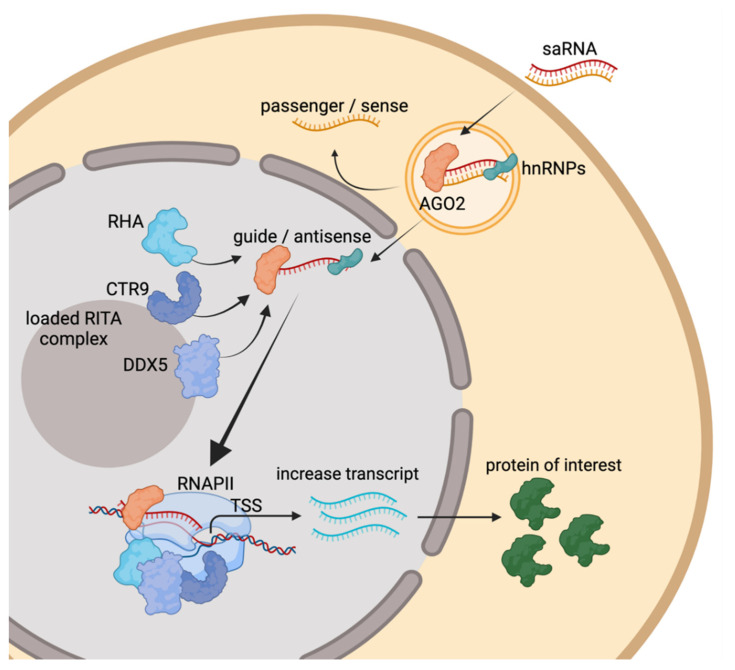
Molecular pathway for RNA activation. saRNA enters the cell and interacts with AGO2 (Argonaute 2) and hnRNPs in the cytoplasm The dsRNA is unwound, and the sense strand is displaced. The RNA-protein complex enters the nucleus and interacts with RHA and CTR9, forming the RITA complex. RITA activates the transcription of the target gene leading to an increase in target transcript and protein of interest. TSS—transcription start site.

saRNA is commonly defined as chemically synthesized 21nt-length small double stranded RNA (dsRNA) oligonucleotides that can positively and reversibly upregulate their target genes above endogenous level. While many other mechanisms of RNA-mediated transcriptional increase have been discovered, this review highlights mechanisms that are strictly mediated by saRNA. The mode of action for saRNA is dependent on the complementarity at the 5′-region of the antisense or guide strand oligo to the intended target DNA near or within gene promoters, this is shown by pull-down and ChIP experiments [12,13]. The 2nd to the 8th nucleotide position at the 5′-end of the guide strand defines the seed region of a saRNA, mutations in this region affect the activity of the saRNA [2,3,12]. Meanwhile, mismatches outside this region are well tolerated even if they occur in consecutive residues [14]. After cellular internalization, the saRNA is recognized in the cytoplasm by dsRNA loading factors and is preferentially loaded into AGO2 protein (Figure 1) [12,15]. Knockout and co-IP experiments using biotinylated saRNA confirmed the role of AGO2 for saRNA activity [16]. Furthermore, AGO2 does not induce cleavage of RNA target complementary to the saRNA, this demonstrates that the ‘slicer activity’ of AGO2 is dispensable for saRNA activity [12,13]. Upon interaction with saRNA, AGO2 discharges one of the two strands of dsRNA, namely the passenger strand, the retained strand is termed guide strand and is also named the antisense sequence. By design, retention of the guide strand is promoted by the addition of modifications to the 5′-end of passenger strand (e.g., inverted abasic) to lower the chances of AGO2 loading [13]. Several independent researchers reported the involvement of AGO1 in the mechanism of action of saRNA using ChIP analysis or siRNA-mediated knockdown [15,17,18,19]. This suggests that some saRNA might preferentially use other members of the AGO family (AGO1, 3 and 4) for their activity.

Several heterogeneous nuclear ribonucleoproteins (hnRNPs), in particular, hnRNPA2/B1 are required for the saRNA mechanism of action [16,20]. A complex consisting of guide RNA, hnRNPs and AGO2 is imported into the nucleus where it binds directly to DNA and facilitates the assembly of an RNA-induced transcriptional activation (RITA) complex (Figure 1). RITA is made up of RNA helicase A (RHA), RNA polymerase-associated protein CTR9 homolog (part of the PAF1 complex) and DEAD-box helicase 5 (DDX5). This complex interacts with RNA polymerase II (RNAPII) to initiate transcription and to ensure productive elongation of the mRNA [13,16,21]. This process involves nucleosome repositioning and the formation of nucleosome-depleted regions on genomic locations such as the TATA box, CpG islands, proximal enhancers, and proximal promoters. Exposure of these regulatory binding sites to facilitate the binding of RNAPII to the transcription start site for the assembly of the transcription preinitiation complex [22]. At the chromatin level, saRNA activation has been reported to cause reduction of acetylation at histones H3K9 and H3K14, increased di/trimethylation at histone H3K4, reduced dimethylation of H3K9, increased dimethylation of H3K4 and monoubiquitination of H2B [2,19,23,24]. The PAF1 complex is crucial in initiating these histone modifications by recruitment of key histone-modifying factors to the RNAPII complex. In some instances, instead of binding to the complementary DNA sequences, saRNAs could alternatively increase the target transcript by binding to promoter-associated nascent transcripts or long non-coding RNAs in sense and/or antisense orientation and favors epigenetic changes within the promoter regions [15,21].

## 3. Applications of saRNA in Preclinical Disease Models

Many publications and patents have tested and documented the activity of saRNA in vitro. Dar et al. created a database that collects information of saRNA tested in cell culture available at http://bioinfo.imtech.res.in/manojk/sarna/ (accessed 28 October 2021) [25]. For in vivo validations, several studies have shown saRNA therapeutic efficacy in animal disease models. In the context of cancer, the most popular targets are tumor suppressor genes and of those, proteins from the p53-p21 axis are the most extensively studied targets.

dsP21-322 is a saRNA designed to increase p21 expression [26] and has shown a reduction in tumor growth in several pre-clinical cancer models such as prostate, lung or pancreatic carcinomas [27,28,29]. Local delivery of lipidic particles containing p21 saRNA to orthotopic models of bladder and colorectal cancer also controlled tumor growth [30,31] In a similar manner, targeting p53 activation in pheochromocytoma resulted in significant tumor reduction in athymic nude mice [32]. It is worth noting that while these xenograft models are very useful in evaluating the efficacy of the compound tested, the lack of a fully functional immune system makes them difficult to translate into a clinical setting. Additionally, most of these studies were performed using intratumor delivery of the encapsulated RNAs to cancer cells, which overlooks the effect of immune system recruitment to the site of disease.

Notch signaling can be either oncogenic or tumor suppressive depending on the context. It has been described as suppressive for various tumor models, including squamous and small-cell lung carcinomas, brain and liver cancer [33]. Using a castration-resistant prostate cancer cell line, Ma and colleagues showed that Notch1 activation increased apoptosis while reduced survival, migration, invasion and in vitro angiogenesis [34]. These phenotypes correlated with a certain degree of tumor growth reduction in vivo. Mechanistically, one interesting observation was that an increase in the expression of E-cadherin limited epithelial-to-mesenchymal transition (EMT). Consistent with this hypothesis, studies designed to activate E-cadherin using saRNA have also shown anti-cancer effects in models of renal and breast carcinomas [35,36].

As mentioned previously, it is not always feasible to extrapolate murine tumor models into the clinical setting due to the more complicated nature of human studies and that immune system effects are bypassed in those experiments. An alternative strategy to identify saRNA target genes would be to extract data from human disease databases and reverse-translate it into the pre-clinical setting. This strategy was adopted by Li and colleagues who identified ARGLU1 as a potential therapeutic target for gastric cancer. In this study, a nine-gene gastric cancer signature was identified by the bioinformatic analysis of several gastric cancer databases. Among those selected, saRNA for ARGLU1 showed efficacy in reducing tumor growth in patient-derived organoids and xenografts [37].

To date, activating RNA strategies have been mainly applied in cancer research, as they are known to be safe, cost-effective and have the capacity to target previously undruggable genes in a wide array of cell types [38]. Therefore, their application is not restricted to the field of oncology. For example, FOXG1, which is a key regulator of cortico-cerebral development and is implicated in neuronal differentiation. In humans, FOXG1 allele dosage is crucial to neurological health, which makes it an ideal platform for saRNA technology. FOXG1 saRNA enhanced FOXG1 expression in vitro and in vivo, leading to improved neural development. Interestingly, this work suggested the involvement of AGO1 as a key player for gene activation in contrast to the general assumption that AGO2 is the main driver of RNAa-dependent gene activation [17]. Due to their nature, monogenic disorders also constitute an attractive target for activating RNA technologies. For example, familiar hypercalciuric nephrolithiasis is a disease leading to kidney stone formation that results in acute pain and renal insufficiency [39]. saRNA mediated activation of TRPV5, an ion channel that plays a crucial role in calcium regulation in the kidney, has been shown to reduce urinary calcium excretion and calcium crystals deposition in vivo [40].

## 4. saRNA Targeting CEBPA Expression Demonstrates Anti-Tumor Activity

The most advanced therapeutic saRNA research program is the upregulation of the CEBPA gene for the treatment of solid tumors. CEBPA is a transcription factor governing a wide array of cellular processes. Downregulation of CEBPA has been implicated in leukemia [41] and in many solid tumors including liver, breast, lung and prostate cancer (reviewed in [42]). To restore CEBPA expression, MiNA Therapeutics has developed CEBPA-51 saRNA, a double-stranded RNA oligo duplex to specifically upregulate the transcription of the CEBPA gene, several 2′-*o*-methyl modified bases were incorporated into the oligo to avoid non-specific immune stimulatory activity [13]. This saRNA was tested using several delivery systems and has shown anti-tumor efficacy in different preclinical settings. CEBPA is a tumor suppressor and several mechanisms of CEBPA downregulation in cancer have been reported [43,44,45]. Reconstitution of CEBPA expression by plasmid transfection or lentiviral transduction inhibited proliferation and migration of cancer cells and suppressed tumor growth and metastasis in vivo [46,47,48]. Similarly, it has been reported that upregulation of CEBPA expression using saRNA slows the growth of rapidly proliferating hepatoma cells [13,49]. Delivery of the CEBPA saRNA by means of dendrimer to cirrhotic rat model with multifocal liver tumors reduces tumor burden and improves liver function [49]. Tumor suppressive effects of CEBPA saRNA are also demonstrated in preclinical models of pancreatic ductal adenocarcinoma. In these studies, CEBPA saRNA was conjugated to RNA aptamers that showed high binding affinity and were efficiently internalized into target PANC-1 cells. Treatment of subcutaneous pancreatic mouse tumor model or the advanced liver-metastatic pancreatic cancer model with the CEBPA-saRNA aptamer conjugate resulted in a significant reduction in tumor growth, mediated by the increase of p21 gene which is downstream of CEBPA transcriptional activation [50,51]. In vitro model has demonstrated that it is possible to combine siRNA activity with saRNA activity and the CEBPB gene which has opposing activity from CEBPA was tested in this study. CEBPB promotes tumorigenesis in many tissues through altering cytokine/chemokine expression and mediating resistance to apoptosis [52]. The combination of CEBPB siRNA with CEBPA saRNA in transfection experiments resulted in a more pronounced reduction in the proliferation of cancer cell lines [16].

## 5. Formulated CEBPA-51 saRNA (MTL-CEBPA) Targets Immunosuppressive Myeloid Cells and Synergize with Other Anti-Cancer Agents

To progress CEBPA-51 saRNA into clinical trials, liposomal nanoparticles (SMARTICLES) was selected as the delivery vehicle. MTL-CEBPA, the clinical candidate is produced by formulating CEBPA-51 saRNA in SMARTICLES. Preclinical efficacy of MTL-CEBPA was first demonstrated in rat liver diseases, treatment with MTL-CEBPA resulted in the increase of CEBPA expression in the liver and this promotes disease reversal of several models including the DEN-induced cirrhotic hepatocellular carcinoma (HCC), carbon-tetrachloride-induced fibrosis and the methionine and choline-deficient diet-induced non-alcoholic steatohepatitis [53].

CEBPA is a master regulator of myeloid differentiation [54]. Mice with knock-out of CEBPA have an increase in the number of myeloid-derived suppressor cells (MDSC) in the tumor, increased expression of proangiogenic markers and accelerated tumor growth [55]. An earlier study demonstrated that peritoneal macrophages collected from tumor-bearing mice have diminished CEBPA expression levels and have marked reduction of IL12 production upon stimulation—a phenotype that is consistent with MDSC immunosuppressive characteristics [56]. MTL-CEBPA is highly suited to restore CEBPA expression in MDSC. Intravenous injection of a fluorescently labeled MTL-CEBPA in a rodent model showed biodistribution of the compound to macrophages and dendritic cells in the peripheral blood, analysis of myeloid cells within organs and tumors tissue also indicated substantial uptake of the labeled compound. An increase in CEBPA expression was confirmed in monocytic-MDSC (M-MDSC) and tumor-associated macrophage (TAM) populations isolated from the tumors of mice treated with MTL-CEBPA. These cells also confirmed a reversal of their immunosuppressive function in ex vivo T cell co-culture experiments [57].

As profound immune suppression mediated by MDSC and TAM within the tumor microenvironment greatly limits the response rate of patients undergoing immune checkpoint inhibitors (ICI) treatment, it is important to test the effect of ICI in combination with MDSC alternating agents [58]. A comprehensive series of preclinical combination studies involving MTL-CEBPA with ICI is reported. MTL-CEBPA improved the anti-tumor effect of PD-1 antibody and CTLA4 antibody treatment in murine models of colon and lung carcinoma, respectively [57,59]. Furthermore, since combinations of MTL-CEBPA and CPI are well-tolerated, further cancer treatment modalities could be added to maximize the anti-tumor potential of these immunotherapy-based combinations. Cancer cells produce prostaglandin E2 (PGE2) through the PTGS2-COX2 pathway to effect immune evasion by several mechanisms [60]. Celecoxib, a selective COX-2 inhibitor that suppresses cancer cell proliferation [61] was applied with a combination of CTLA4 antibody and MTL-CEBPA. While monotherapy or a combination of any of the two modalities did not fully suppress the growth of a CPI resistant Lewis lung carcinoma rodent model, combining the three therapies completely inhibited tumor growth and tumors from this treatment group were significantly smaller than groups treated with any of the two combinations of compounds [57]. Besides small molecule therapeutics, the combination of MTL-CEBPA and PD-1 antibody was also tested with radiofrequency ablation (RFA), a standard treatment option for HCC in the clinic associated with a significant survival benefit [62]. In this study, subcutaneous tumors were implanted on both flanks and RFA treatment was administered to only one of the flanks. Treatment of MTL-CEBPA with PD-1 antibody synergized with the effects of RFA and led to strong inhibition of the non-RFA treated tumor on the other flank. The abscopal effect observed was indicative of an immunomodulatory effect mediated by combining MTL-CEBPA, PD-1 antibody with RFA, this allowed priming of T cells that inhibited the growth of distal tumors. Indeed, these tumors exhibited the highest level of tumor-infiltrating cytotoxic T cells and natural killer T cells [59]. Other anti-cancer agents that were tested and demonstrated enhanced efficacy when applied in combination with MTL-CEBPA included sorafenib (to be discussed in the next section) [63] and Lipofermata, an inhibitor of PMN-MDSC [57,64].

**Figure 2:**
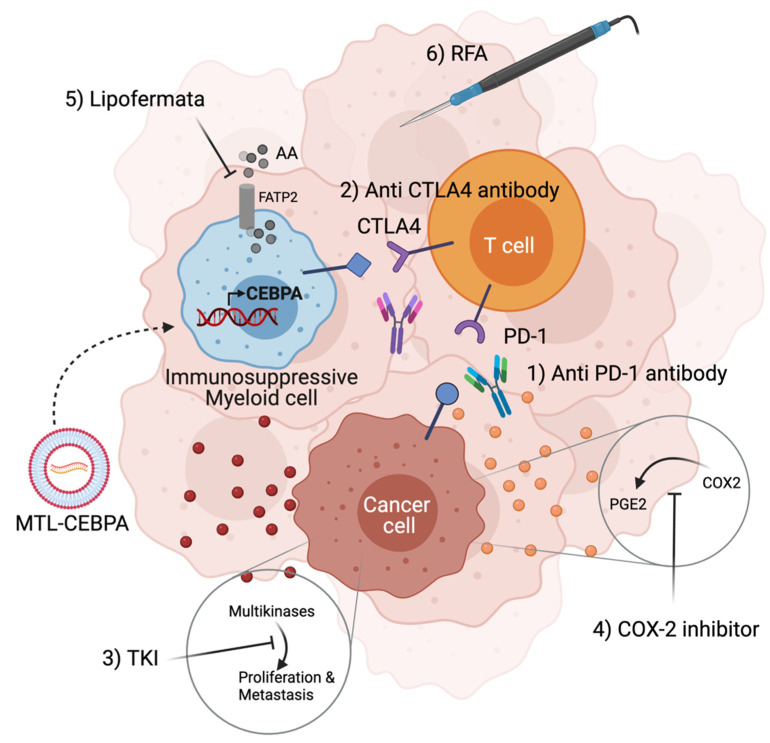
MTL-CEBPA synergizes with other anti-cancer therapy to inhibit tumor growth in preclinical tumor models. MTL-CEBPA inhibits the immunosuppressive function of myeloid cells within the tumor and improves the efficacies of T-cell targeting therapy (1,2), cancer cell inhibitory therapy (3,4), PMN-MDSC inhibitory therapy (5) and radiofrequency ablation (6). Triple combination studies consisting of MTL-CEBPA with anti-PD-1 antibody and RFA or MTL-CEBPA with anti-CTLA4 antibody and COX-2 inhibitor show further synergistic effects.

Unlike monogenic disorders, the tumor microenvironment is an ecosystem comprising many different cell types expressing an array of factors to support uncontrollable growth. Preclinical studies of MTL-CEBPA established saRNA therapeutics as a safe and well-tolerated modality that can be combined with other drugs to produce an all-round approach to tumor control. Key cellular components within the tumor microenvironment are myeloid cells, T-cells and cancer cells. MTL-CEBPA reduces the immunosuppressive activity of myeloid cells to allow better efficacies of compounds targeting other cell types (Figure 2).

## 6. Promising Observations of MTL-CEBPA in Clinical Trials

A Phase 1a, open-label, dose-escalation trial of MTL-CEBPA was conducted in patients with advanced HCC. The administration of MTL-CEBPA demonstrated safety and a potential synergistic efficacy with tyrosine kinase inhibitor (TKI). Seven patients discontinued from MTL-CEBPA treatment were subsequently treated with TKIs including regorafenib, lenvatinib and sorafenib. Three of these patients who were TKI naïve showed a complete radiological tumor response and one patient had a partial response. Furthermore, two of the patients with lung metastases had complete resolution of multiple lung lesions [65]. The observed effects are most likely due to the combination of MTL-CEBPA with sorafenib as the original Phase 3 study of sorafenib, reported only two partial responders and no complete responder out of 299 patients randomized to receive the drug [66]. As immunosuppressive myeloid cells are the most likely cause of TKI resistance in HCC models [67,68], prior treatment with MTL-CEBPA is thought to reverse this suppression to allow for better efficacy of the TKI treatment.

Based on these observations, MTL-CEBPA entered a Phase 1b of the clinical trial in combination with sorafenib which was given either concomitantly or sequentially. Radiological regression of tumor was observed in 26.7% of patients with underlying viral (HBV or HCV) etiology and three of these patients developed a complete response. Analysis of peripheral blood from these patients showed a decrease in the MDSC population, while post treatment liver biopsy analysis of a patient who had complete response showed downregulation of the pro-tumor M2 tumor-associated macrophages [57]. These results prompted the initiation of a large international multi-center study of MTL-CEBPA in combination with pembrolizumab (PD-1 antibody) in adult patients with advanced solid tumors.

## 7. Future Perspective for saRNA Therapeutics

Since the initial report of RNAa activity and coining of the term saRNA in 2006, the research community has actively explored possibilities for utilizing these small 21 nucleotide dsRNAs to achieve transcriptional increase of native genes for therapeutic purposes. While most existing therapies including small molecules, antibodies, ASO and siRNA inhibit target activity, saRNA represents a new treatment that could directly activate signaling pathways [69]. The exciting potential of saRNA therapeutics is evident from the large volume of research showing efficacy in preclinical models and is further cemented by the promising observations reported by MTL-CEBPA in clinical trials [57,70].

As a relatively new treatment modality, optimizations to improve saRNA therapeutics and the range of diseases that they can treat are being explored. Delivery of saRNA to the desired organ and cell type are under investigation using lipid nanoparticles, lipid and polymer hybrids, dendrimers, aptamers and GalNac as delivery vehicles [71,72]. The potency of saRNA can be augmented from a deeper understanding of saRNA biology, smarter algorithm-based sequence selection and improvement of oligo stability by the addition of chemical modifications. The combined output of these optimizations will lead to the invention of a new generation of saRNA therapeutics to further rescue the downregulation of previously undruggable gene targets.
